# Compliance and treatment satisfaction of post menopausal women treated for osteoporosis. Compliance with osteoporosis treatment

**DOI:** 10.1186/1472-6874-10-26

**Published:** 2010-08-20

**Authors:** Dominique Huas, Françoise Debiais, Francis Blotman, Bernard Cortet, Florence Mercier, Chantal Rousseaux, Véronique Berger, Anne-Françoise Gaudin, François-Emery Cotté

**Affiliations:** 1 Department of General Practice, UFR Paris 7, Paris, France; 2 Rheumatology Department, Poitiers University Hospital, Poitiers, France; 3 Rheumatology Department, Montpellier University Hospital, Montpellier, France; 4 Rheumatology Department, Roger Salengro Hospital, Lille, France; 5 STAT-Process, Port-Mort, France; 6 Nukléus, Paris, France; 7 Laboratoire GlaxoSmithKline, Marly le Roi, France; 8 CERMES, IFR69, INSERM U750, National Institute of Health and Medical Research, Villejuif, France

## Abstract

**Background:**

Adherence to anti-osteoporosis treatments is poor, exposing treated women to increased fracture risk. Determinants of poor adherence are poorly understood. The study aims to determine physician- and patient- rated treatment compliance with osteoporosis treatments and to evaluate factors influencing compliance.

**Methods:**

This was an observational, cross-sectional pharmacoepidemiological study with a randomly-selected sample of 420 GPs, 154 rheumatologists and 110 gynaecologists practicing in France. Investigators included post-menopausal women with a diagnosis of osteoporosis and a treatment initiated in the previous six months. Investigators completed a questionnaire on clinical features, treatments and medical history, and on patient compliance. Patients completed a questionnaire on sociodemographic features, lifestyle, attitudes and knowledge about osteoporosis, treatment compliance, treatment satisfaction and quality of life. Treatment compliance was evaluated with the Morisky Medication-taking Adherence Scale. Variables collected in the questionnaires were evaluated for association with compliance using multivariate logistic regression analysis.

**Results:**

785 women were evaluated. Physicians considered 95.4% of the sample to be compliant, but only 65.5% of women considered themselves compliant. The correlation between patient and physician perceptions of compliance was low (κ: 0.11 [95% CI: 0.06 to 0.16]). Patient-rated compliance was highest for monthly bisphosphonates (79.7%) and lowest for hormone substitution therapy (50.0%). Six variables were associated with compliance: treatment administration frequency, perceptions of long-term treatment acceptability, perceptions of health consequences of osteoporosis, perceptions of knowledge about osteoporosis, exercise and mental quality of life.

**Conclusion:**

Compliance to anti-osteoporosis treatments is poor. Reduction of dosing regimen frequency and patient education may be useful ways of improving compliance.

## Background

Anti-osteoporosis treatments such as bisphosphonates, selective oestrogen receptor modulators (SERMs) and strontium ranelate have been demonstrated to reduce significantly the risk of osteoporotic fracture in women with post-menopausal osteoporosis [1]. Nonetheless, the effectiveness of these treatments in routine clinical practice may be compromised by poor treatment adherence. Indeed, a number of studies have reported low compliance or persistence rates, notably with bisphosphonates [2], and others have demonstrated that poor adherence compromises control of fracture risk [3,4].

A number of strategies have been proposed for improving adherence to treatment in post-menopausal osteoporosis, including reduction of dosing frequency, patient education programmes and bone mineral densitometry or other surrogate markers to help patients follow treatment-related changes in bone mass [5]. In order to evaluate the utility of such measures, it is important to acquire data on how patients view their own adherence to treatment and on the different patient variables that are associated with adherence.

We have recently performed a large, observational, pharmacoepidemiological study of osteoporosis and its treatment in primary and secondary care in France (POSTEPI study). The primary objective of the study was to describe the characteristics of women receiving treatment for osteoporosis diagnosed in the previous six months. Secondary objectives were to identify variables potentially associated with different treatment regimens, to assess impact on quality of life, and to evaluate patient adherence to, and satisfaction with, their anti-osteoporosis treatment. The treatment data will be presented elsewhere. This article reports the data on adherence and patient satisfaction.

## Methods

This was an observational, cross-sectional pharmacoepidemiological study performed in France between November 2007 and March 2008.

### Participating physicians

General practitioners (GPs), gynaecologists and rheumatologists participated in the study. These were selected at random from a national physician list (CEGEDIM database) using a sampling method stratified by region. The planned number of participating physician was 650.

### Subjects

Participants included all women in whom bone densitometry had been performed or who had experienced a fracture not related to trauma or cancer in the previous six months in a patient registry. Of these, the first three post-menopausal women in whom a diagnosis of osteoporosis had been made on the basis of low bone mass density or fracture occurrence in the previous six months, and for whom osteoporosis treatment was initiated, constituted, the questionnaire population. Exclusion criteria included participation in studies likely to have influenced treatment and illiteracy.

### Data collection

Participating physicians provided general professional information and specific information on osteoporosis management. For each patient included in the registry, the physician noted the age of the patient, the age at menopause, the age at which osteoporosis was diagnosed, information on densitometry, fractures, fracture risk factors and any current or planned osteoporosis treatments. For the questionnaire population, each participating physician completed a medical questionnaire. This included items on height, weight, exercise, fracture history, osteoporosis management, comorbidities, and comedication. In addition, the physician provided patients with a questionnaire to complete. This collected data on sociodemographic features, lifestyle, attitudes and knowledge concerning osteoporosis and its treatment, treatment compliance, treatment satisfaction and quality of life. Information on compliance was collected both from physicians and from patients. Physicians were asked whether they considered their patients to be fully compliant. Treatment compliance from the patient point of view was evaluated with the French version of Morisky Medication-taking Adherence Scale (MMAS) (Additional file 1) [6] and quality of life with the SF-12 health profile measure, both completed by the patient. Both were used in their validated French translations. Treatment satisfaction was assessed by asking the patient if she was satisfied with her osteoporosis treatment. Five response modalities were possible. For the purposes of the analysis, replies were grouped into three classes (very/rather unsatisfied, neither satisfied nor dissatisfied, or very/quite satisfied). Patients were also asked whether they considered their treatment regimen to be adapted to their lifestyle and whether they considered the frequency of administration of their treatment regimen to be easy to maintain over the long term. The completed questionnaire was returned directly to the data management centre.

The MMAS contains four items to which a yes or no reply is given (Additional file 2). These questions are '*Do you ever forget to take your [osteoporosis] medicine?*', '*Do you ever have problems remembering to take your [osteoporosis] medication?*', '*When you feel better, do you sometimes stop taking your [osteoporosis] medicine?*' and '*Sometimes, if you feel worse when you take your [osteoporosis] medicine, do you stop taking it?*'. Each 'no' reply is scored as one and each 'yes' reply is scored as zero, allowing a total possible score ranging from zero (worst compliance) to four (full compliance). A patient was considered non-compliant to treatment if her score on the MMAS was less than four.

### Statistical analysis

The present analysis was restricted to those patients in whom a treatment had been initiated during the previous six months. Statistical comparisons were performed using the χ^2 ^test or Fisher's exact test for categorical variables and analysis of variance or the Wilcoxon test for quantitative variables. All tests were two-tailed. A probability threshold of 0.05 was taken as statistically significant. Variables associated with compliance were evaluated by multivariate logistic regression analysis using a rising stepwise procedure with a cut-off probability threshold of 0.1 at each step. The variables entered into this analysis corresponded to all those collected in the case report form whose frequencies differed between compliant and non-compliant patients at a probability level of 0.2 in univariate analysis. A final multivariate model was generated in which only variables retained in the stepwise model were entered in order to generate odds ratios for the association with compliance. Data were analysed using SAS^® ^software, Version 8.2 (SAS, Cary, USA) on Windows.

### Ethics

The survey protocol was submitted for evaluation to the CCTIRS (National Ethics Advisory Board). They considered that participation of patients in the study would not affect their medical care, and therefore that it was not necessary to obtain formal Ethics Committee approval nor to collect signed informed consent from each patient. The only requirement stipulated was that formal information on the goals and methods of the study be provided for each patient. Procedures for data collection and management were approved by the Conseil National de l'Informatique et des Libertés (CNIL), which ensures that all medical information is kept confidential and anonymous.

## Results

### Participating physicians

Overall, 684 physicians included patients in the registry, namely 420 GPs, 154 rheumatologists and 110 gynaecologists.

### Subjects

The first three registry patients included per investigator (n = 1,306) were entered into the questionnaire study and proposed an autoquestionnaire. Completed autoquestionnaires were received from 1,217 women (93.2%), who constituted the autoquestionnaire population. The analysis reported here is restricted to the 785 women who had already been prescribed a treatment at the time of the consultation. For the remaining 521 women, treatment was initiated during the consultation and compliance thus could not be assessed.

Patient characteristics and treatment are presented in Table 1.

**Table 1:** Characteristics of women treated for osteoporosis at the time of the consultation (questionnaire population; N = 785).

Patient characteristics		Treatments	
Age (years)	66.30 ± 9.07	Daily bisphosphonates	17 (2.2%)
Age at menopause (years)	49.75 ± 3.97	Weekly bisphosphonates	305 (40.6%)
Time since menopause (years)	16.49 ± 9.38	Monthly bisphosphonates	202 (26.9%)
Densitometry in the last six months	667 (85.3%)	SERMs	76 (10.1%)
Osteoporotic fractures	389 (51.7%)	Strontium ranelate	119 (15.8%)
At least one risk factor	618 (78.7%)	Others	26 (3.5%)

Data are presented as mean ± SD for quantitative variables and as numbers of women (%) for categorical variables. Data were missing for up to six patients for certain variables. SERM: selective oestrogen receptor modulators. 'Others' include parathyroid hormone analogues and hormone substitution therapy.

### Compliance

**Figure 1:**
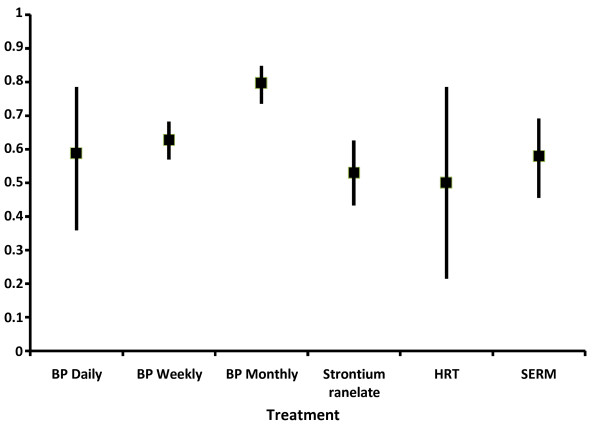
**Proportion of patients rating themselves as fully compliant (MMAS scale score = 4) according to medication class**. Data presented with 95% Confidence Interval limits (95% C.I.). BP: bisphosphonate; HRT: hormone replacement therapy; SERM: selective oestrogen receptor modulator.

The physicians considered their patients to be fully compliant in almost all cases (Table 2). From the patients' perspective, 65.5% of women considered themselves to be fully compliant (MMAS score = 4), and the correlation between patient and physician perceptions of compliance was very poor (*p *= <0.001; MacNemar test; κ coefficient = 0.11 [95% CI: 0.06 to 0.16]). Nonetheless, patients who rated themselves as fully compliant were more likely to be considered compliant by their physicians and *vice versa *(*p *< 0.01: Cochran-Mantel Haenszel test; Table 2). Patient-rated compliance differed between the different treatments (p <0.001; Figure 1), being highest for monthly bisphosphonates (79.7%) and lowest for hormone substitution therapy (50.0%).

**Table 2:** Compliance with treatment as rated by the physician and patient.

	TOTAL
***Physician-rated compliance***	
Patients considered compliant	748 (95.4%)

***Patient-rated compliance ***	
MMAS score (mean ± SD)	3.34 ± 1.07
Compliant patients (Morisky score = 4)	483 (65.5%)

***Concordance***	
Patients considered compliant by both physician and patient	476 (64.6%)
Patients considered non-compliant by both physician and patient	26 (3.5%)
Kappa concordance coefficient	0.11 [95% CI: 0.06 to 0.16]

Significant differences in reply distributions were not observed between the three physician groups. Data for physician-reported compliance concern the questionnaire population (N = 785; missing data: N = 1) and for patient-rated compliance and concordance the autoquestionnaire population (N = 751; missing data: N = 13). MMAS: Morisky Medication-taking Adherence Scale.

### Satisfaction

Data on treatment satisfaction is presented in Table 3. The rate of treatment satisfaction varied with the frequency of administration (*p *= 0.001; χ^2 ^test), being highest with monthly treatments. Satisfaction did not however, differ between the different classes of medication (*p *= 0.05; χ^2 ^test; data not shown).

**Table 3:** Treatment satisfaction as a function of frequency of administration (all treatments combined).

	Daily	Weekly	Monthly	TOTAL
***Patient satisfaction regarding osteoporosis treatment***				*p *≤ 0.001
Very unsatisfied or rather unsatisfied	4 (2%)	5 (8%)	1 (4%)	10 (13.8%)
Neither satisfied nor dissatisfied	081 (38.9%)	119 (41.2%)	069 (30.5%)	269 (37.2%)
Quite satisfied or very satisfied	123 (59.1%)	165 (57.1%)	156 (69.0%)	444 (61.4%)

***Treatment regimen considered adapted to lifestyle***				
	188 (89.5%)	279 (97.2%)	226 (99.6%)	*p *≤ 0.001

***Frequency of treatment administration considered easy to maintain over the long-term***				
	170 (81.3%)	262 (91.3%)	218 (96.5%)	*p *≤ 0.001

Data were missing for up to three patients for certain variables.

### Variables associated with compliance

In a first step, all variables collected during the course of the study were evaluated for their potential association with patient-reported compliance (MMAS score = 4 compared to score < 4) using univariate analysis. Variables related to attitudes to disease and treatment identified on the autoquestionnaire are listed in Table 4. After a stepwise multivariate regression analysis, six variables were retained and corresponding odds ratios generated (Table 5), namely frequency of treatment administration, whether the patient considered the frequency of treatment administration to be adapted to long-term treatment, whether she considered that her osteoporosis could have consequences for her health, whether she considered herself well-informed about osteoporosis, whether she walked for at least twenty minutes each day and the mental component score of the SF-12.

**Table 4:** Autoquestionnaire variables independently associated with compliance identified by univariate regression analysis.

Variable	Response modality	Non-compliant N = 255	Compliant N = 483	*p*
Osteoporosis considered as an illness	Yes	172 (67.1%)	378 (79.1%)	0.001

Consequences of osteoporosis according to the patient	Serious Quite serious Not serious	62 (24.3%) 150 (58.8%) 43 (16.9%)	198 (41.3%) 244 (50.8%) 81 (11.0%)	≤0.001

Known osteoporotic risk factors before diagnosis	Yes	43 (16.9%)	143 (29.6%)	≤0.001

Well Informed on osteoporosis since diagnosis	Strongly agree Agree Disagree Strongly disagree	41 (16.1%) 46 (18.1%) 164 (64.6%) 3 (1.2%)	170 (35.2%) 37 (7.7%) 274 (56.7%) 2 (0.4%)	≤0.001

Osteoporosis treatment considered as a priority	Strongly agree Agree Disagree Strongly disagree	69 (27.1%) 37 (14.5%) 144 (56.5%) 5 (2.0%)	228 (47.3%) 35 (7.3%) 216 (44.8%) 3 (0.6%)	≤0.001

Patients perspective: possible	Less than 3 months 3 months to 1 year 1 to 3 years More than 3 years	1 (0.4%) 9 (3.7%) 68 (27.9%) 166 (68.0%)	0 (0.0%) 4 (0.8%) 96 (20.1%) 377 (79.0%)	≤0.001

Walking over 20 min per day	Yes	128 (50.2%)	312 (65.0%)	≤0.001

Physical component score of SF12 (PCS)	Score unit	44.50 ± 7.66	45.85 ± 8.06	0.041

Mental component score of SF12 (MCS)	Score unit	43.16 ± 9.78	46.03 ± 10.06	≤0.001

Satisfied by osteoporosis treatment	Very/rather unsatisfied Neither satisfied nor unsatisfied Very/quite satisfied	6 (2.4%) 130 (51.2%) 1180 (46.4%)	4 (0.8%) 141 (29.6%) 331 (69.5%)	≤0.001

Treatment regimen adapted to your lifestyle	Yes	235 (92.5%)	468 (97.5%)	0.003

Treatment regimen difficult to follow for a long-time	No	201 (79.8%)	461 (96.0%)	≤0.001

**Table 5:** Odds ratios variables independently associated with compliance identified by multivariate regression analysis (odds ratios presented with their 95% confidence intervals).

Effect	Response	Odds Ratio	*p*
Frequency of administration adapted to long-term treatment	No	1.00	
	Yes	**4.227 [2.272; 7.863]**	**< 0.0001**
Frequency of treatment administration	Daily	1.00	.
	Weekly	1.273 [0.836; 1.938]	0.2607
	Monthly	**2.232 [1.367; 3.643]**	**0.0013**
Consequences of osteoporosis according to the patient	Serious	1.00	
	Quite serious	**0.617 [0.407; 0.937]**	**0.0234**
	Not serious	**0.466 [0.250; 0.868]**	**0.0161**
Well Informed on osteoporosis since diagnosis	Strongly agree	1.00	.
	Weakly agree	**0.564 [0.358; 0.889]**	**0.0006**
	Disagree	**0.322 [0.169; 0.616]**	**0.0136**
Walking over 20 minutes a day	No	1.00	
	Yes	**1.496 [1.031; 2.171]**	**0.0341**
Mental component score of SF12	One point increment	**1.021 [1.002; 1.040]**	**0.0328**

## Discussion

In this study, we observed a large discrepancy between treatment compliance as evaluated by the investigator and as considered by the patient. Although the physicians thought that compliance was adequate for over 95% of women, only two-thirds of the patients rated themselves as fully compliant.

Compliance rates (as defined by the patient) differed between medication groups, being highest for bisphosphonates and lowest for HRT and strontium ranelate. Given the very small number of women taking HRT, the precision of the compliance rate for this treatment should be regarded as limited. Among the bisphosphonates, significant differences in compliance were observed between the various treatment regimens, being highest for monthly preparations and lowest for daily regimens. Indeed, in the multivariate regression analysis, treatment administration frequency was the variable with the strongest association with compliance, with an increased probability of being compliant to a monthly treatment over twofold higher than for a daily treatment. Other variables associated with improved compliance were the patient's view of the consequences of osteoporosis, knowledge about osteoporosis, walking over twenty minutes a day, and a higher mental component quality of life score. Somewhat surprisingly, previous fracture experience did not influence compliance. It will be important to validate whether these determinants of compliance can be reproduced in prospective longitudinal studies of the treatment of osteoporosis.

Around sixty percent of women reported being quite or very satisfied with their treatment, and a similar proportion considered following their treatment to be a priority for their health. Treatment satisfaction was also higher in women receiving a monthly treatment than a weekly treatment, and in those receiving a weekly treatment compared to a daily one.

The study has a number of strengths and limitations. The strengths include the relatively large sample size, the sampling method, which should ensure representativity of women consulting for osteoporosis in primary and secondary care in France, the relatively high response rate, and the collection of data directly from the patients. The principal limitation relates to the choice of the instrument for determining compliance to treatment. The use of a patient-completed questionnaire is appropriate for a naturalistic study such as this, but relies on the accuracy of patient self-report, which cannot be independently controlled. This potential source of bias is compounded by the fact that the data were collected retrospectively. The use of a more objective measure, such as pill count, may have provided more accurate data but could have introduced a significant uncontrolled bias in that implementation of the measure may have modified the compliance behaviour that it was set up to report. The MMAS [6] was originally developed to assess compliance to medication use in patients with essential hypertension, but has subsequently been used successfully in other fields of medicine in which adherence is a particular issue, including asthma, HIV, diabetes mellitus and bipolar disorder. In addition, the interpretation of the magnitude of difference between physician- and patient-rated compliance should be tempered by the fact that the instruments used to collect this information were different. In addition, it is clear that other potential determinants of compliance may exist, on which data were not collected in this study. These include patient preference for different treatments and participation in the choice of treatment. Finally, the fact that participation in the study by physicians was voluntary may introduce bias if these are not representative of the French physician population, for example with respect to providing information on treatments to their patients.

Poor discernment by physicians of their patients' subjective view of their medical condition has been demonstrated previously for health outcomes, such as health-related quality of life [7], treatment tolerability [8] or functional disability [9]. With regard to adherence, differences in physician- and patient-perceived compliance such as those described here have been reported previously in many other areas of medicine, notably in psychiatry [10,11]. Similarly, a previous study has shown that physicians did not correctly estimate patient-reported compliance to HIV therapy in 35% of patients [12].

The differences observed in compliance between treatment classes is also consistent with a number of previous studies [13-15], as is the association between compliance and poor quality of life [16]. A relationship between frequency of treatment administration and treatment preference has previously been reported in the two BALTO studies, which used a randomised, cross-over design to compare a monthly and a weekly formulation of bisphosphonates [17].

A recent study reported that many individuals who suffered from a fragility fracture did not associate their fracture with osteoporosis and speculated that there may be a relationship between risk perception and adherence to therapy [18]. Our findings showed that patients in the non-compliant group were characterised by less appropriate perceptions of disease status and less knowledge about risk factors and consequences of osteoporosis. Similarly, not considering osteoporosis to be a serious disease was another factor associated with non-compliance.

The findings of this study have several implications. Firstly, they emphasise the importance of collecting data on adherence directly from the patient, rather than from the physician. Secondly, our results showed that patients who considered themselves poorly informed about their disease reported poor compliance. In a recent Canadian study, osteoporotic women claimed that their healthcare providers did not always give them enough information about medications or gave them information in a format that was difficult to understand. In this study, the absence of a satisfactory exchange of information between physicians and patients was identified as a major determinant of adherence [19]. One of the most effective approaches for improving medication adherence may thus be to encourage more open, co-operative relationships that lead to concordance between the physician and patient [20].

Women who walked over twenty minutes a day also reported better compliance than those who did not, perhaps because they attach more importance to keeping well and taking control of their health in general. The behaviour of such patients who take an active role in managing lifestyle patterns that have an impact on their disease has previously been described in the field of type 2 diabetes [21], where these '*Disease Managers*' tend to show high treatment adherence rates.

The observed association between compliance and patient beliefs and attitudes with respect to osteoporosis reinforces the interest of patient education measures following diagnosis for all women with post-menopausal osteoporosis. Understanding patients' health beliefs better and re-orientating these when they are inaccurate may thus be a useful key to improve compliance [22]. Medication beliefs have indeed been shown elsewhere to be more powerful predictors of reported adherence than clinical and socio-demographic factors [23]. The authors of the latter study proposed that many patients engaged in an implicit cost-benefit analysis in which beliefs about the necessity of their medication are weighed against concerns about the potential adverse effects of taking it, and that these beliefs influenced medication adherence.

Methods to assess adherence are multiple and very different from each other and that, even in the single field. In the osteoporosis literature[24], reported methods based on the care provider (eg, pill count, physical examination for frequent clinical adverse effects), patients (patients' self-report [eg, written or electronic diary], elicited report [eg, global or specific questioning], or other methods), use of devices (eg, pill count by electronic monitoring), biologic elements (eg, serum levels, urine levels, measurement of expected biologic effects), or other methods. Pharmacy data give one measure of adherence whose main advantages are sample size and being almost exempt from selection bias. However, principal well-known limit of pharmacy database is absence of information about patients' behaviors with tablets at their home (omission, drug holiday etc.). In this study, MMAS questionnaire took into account those behavioural aspects as an informative and complementary measure. Thus, our results showing better compliance with monthly regimen are consistent with recent ones on French prescription database [25].

## Conclusions

The POSTEPI study confirmed that compliance to anti-osteoporosis treatments as considered by the patient is poor, but identified reduction of the dosing regimen frequency and patient education measures as potentially useful ways of improving compliance.

## Competing interests

AFG and FEC are employees of Laboratoires GlaxoSmithKline (GSK), France who markets ibandronate and denosumab, two anti-osteoporosis treatments. BC has received consultancy fees from GSK, Laboratoires Roche, Amgen, Novartis and Merck, Sharpe & Dohme with respect to his participation in this and other projects concerning the treatment of osteoporosis. FB, FD and DH have received consultancy fees from GSK and Laboratoire Roche with respect to their participation in this and other projects concerning osteoporosis. FM, CR and VB received stipends from GSK for data analysis (FM) or for coordination of the study (CR and VB).

## Authors' contributions

BC, FB, FD and DH made up the academic steering committee of the study. They advised on the study design, contributed to the analysis of the data and interpretation of the results, recommended the publication policy to follow and contributed to writing and critical revision of the manuscript. FM performed the data management and analysis for the study. CR and VB were responsible for the day-to-day operational management of the study. AFG and FEC conceived the study, recruited the steering committee, oversaw the implementation of the study, and initiated the preparation of the present manuscript. And finally, all authors read and approved the final manuscript.

## Pre-publication history

The pre-publication history for this paper can be accessed here:

http://www.biomedcentral.com/1472-6874/10/26/prepub

## Supplementary Material

Additional file 1 **French version of Morisky Medication-taking Adherence Scale (MMAS) - 4 items**. Linguistic validation by Mapi Research Institute consisted in forward, backward translation, clinician's review and patients' cognitive debriefing. Click here for file

Additional file 2 **US original version of Morisky Medication-taking Adherence Scale (MMAS) - 4 items**. Click here for file
