# Supplementary material for: Compliance and treatment satisfaction of post menopausal women treated for osteoporosis. Compliance with osteoporosis treatment
Source: BMC Womens Health. 2010 Aug 20;10:26. doi: 10.1186/1472-6874-10-26 (PMC2941476; doi:10.1186/1472-6874-10-26)
Supplement: Additional file 1 — French version of Morisky Medication-taking Adherence Scale (MMAS) - 4 items. Linguistic validation by Mapi Research Institute consisted in forward, backward translation, clinician's review and patients' cognitive debriefing. [file 1472-6874-10-26-S1.PDF]

Questionnaire de Morisky sur le respect du traitement (4 questions)

(Cochez une seule réponse par question)

	Oui	*Non*
1. Vous arrive-t-il d'oublier de prendre votre traitement pour (nom de la maladie) ?	❑	❑
2. Avez-vous parfois du mal à vous rappeler de prendre votre traitement pour (nom de la maladie) ?	❑	❑
3. Quand vous vous sentez mieux, vous arrive-t-il d'arrêter de prendre votre traitement pour (nom de la maladie) ?	❑	❑
4. Si vous vous sentez moins bien lorsque vous prenez votre traitement pour (nom de la maladie), arrêtez-vous parfois de le prendre ?	❑	❑

CALCUL DU SCORE

Le MMAS est un questionnaire générique d'évaluation de l'observance thérapeutique rempli par les patients, dans lequel le nom du problème de santé concerné (hypertension artérielle, diabète, cholestérol, sida, contraception, etc.) remplace « nom de la maladie ». Ce questionnaire comporte quatre questions, dont le barème est de 0 pour « Oui » et 1 pour « Non ». Les points pour chaque question sont additionnés pour obtenir un score compris entre 0 et 4.
