# Supplementary material for: Compliance and treatment satisfaction of post menopausal women treated for osteoporosis. Compliance with osteoporosis treatment
Source: BMC Womens Health. 2010 Aug 20;10:26. doi: 10.1186/1472-6874-10-26 (PMC2941476; doi:10.1186/1472-6874-10-26)
Supplement: Additional file 2 — US original version of Morisky Medication-taking Adherence Scale (MMAS) - 4 items. [file 1472-6874-10-26-S2.PDF]

Morisky Medication-Taking Adherence Scale-MMAS (4-item)

English Version

(Please check one box on each line)

	Yes	*No*
1. Do you ever forget to take your (name of health condition) medicine?	o	o
2. Do you ever have problems remembering to take your (name of health condition) medication?	o	o
3. When you feel better, do you sometimes stop taking your (name of health condition) medicine?	o	o
4. Sometimes if you feel worse when you take your (name of health condition) medicine, do you stop taking it?	o	o

MEASUREMENT AND SCORING CRITERIA

The MMAS is a generic self-reported, medication-taking behavior scale in which the specific health issue (high blood pressure, diabetes, elevated cholesterol, HIV, contraception, etc.) is inserted for the "health concern".The MMAS consists of four items with a scoring scheme of "Yes" = 0 and "No" = 1. The items are summed to give a range of scores from 0 to 4.
